# Validation of public health competencies and impact variables for low- and middle-income countries

**DOI:** 10.1186/1471-2458-14-55

**Published:** 2014-01-20

**Authors:** Prisca AC Zwanikken, Lucy Alexander, Nguyen Thanh Huong, Xu Qian, Laura Magana Valladares, Nazar A Mohamed, Xiao Hua Ying, Maria Cecilia Gonzalez-Robledo, Le Cu Linh, Marwa SE Abuzaid Wadidi, Hanan Tahir, Sunisha Neupane, Albert Scherpbier

**Affiliations:** 1 Royal Tropical Institute, Amsterdam, The Netherlands; 2 School of Public Health, University of the Western Cape, Capetown, South Africa; 3 Hanoi School of Public Health, Hanoi, Vietnam; 4 School of Public Health, Fudan University, Shanghai, China; 5 National Institute of Public Health, Cuernavaca, Mexico; 6 Ministry of Health, Khartoum, Sudan; 7 Department of Demography, Hanoi School of Public Health, Hanoi, Vietnam; 8 Human Resource Development, Federal Ministry of Health, Khartoum, Sudan; 9 University of Medical Sciences and Technology, Khartoum, Sudan; 10 Faculty of Health, Medicine and Life Sciences, Maastricht University, Maastricht, The Netherlands

**Keywords:** Public health competencies, Impact, Low- and middle-income countries, Master of Public Health

## Abstract

**Background:**

The number of Master of Public Health (MPH) programmes in low- and middle-income countries (LMICs) is increasing, but questions have been raised regarding the relevance of their outcomes and impacts on context. Although processes for validating public health competencies have taken place in recent years in many high-income countries, validation in LMICs is needed. Furthermore, impact variables of MPH programmes in the workplace and in society have not been developed.

**Method:**

A set of public health competencies and impact variables in the workplace and in society was designed using the competencies and learning objectives of six participating institutions offering MPH programmes in or for LMICs, and the set of competencies of the Council on Linkages Between Academia and Public Health Practice as a reference. The resulting competencies and impact variables differ from those of the Council on Linkages in scope and emphasis on social determinants of health, context specificity and intersectoral competencies. A modified Delphi method was used in this study to validate the public health competencies and impact variables; experts and MPH alumni from China, Vietnam, South Africa, Sudan, Mexico and the Netherlands reviewed them and made recommendations.

**Results:**

The competencies and variables were validated across two Delphi rounds, first with public health experts (N = 31) from the six countries, then with MPH alumni (N = 30). After the first expert round, competencies and impact variables were refined based on the quantitative results and qualitative comments. Both rounds showed high consensus, more so for the competencies than the impact variables. The response rate was 100%.

**Conclusion:**

This is the first time that public health competencies have been validated in LMICs across continents. It is also the first time that impact variables of MPH programmes have been proposed and validated in LMICs across continents. The high degree of consensus between experts and alumni suggests that these public health competencies and impact variables can be used to design and evaluate MPH programmes, as well as for individual and team assessment and continuous professional development in LMICs.

## Background

Responding to the crisis in human resources for health and the need for a well-established public health workforce, the number of Master of Public Health (MPH) training programmes has increased, especially in low- and middle-income countries (LMICs) [1-4]. As the LMIC context differs deeply from High Income Countries, the question has been posed whether existing LMIC programmes equip public health alumni to be effective, and whether the taught competencies from these programmes are relevant to their contexts [4-6]. Since the 1990s, in schooling and higher education globally, detailed descriptions of expected performance or competencies have been commonly used as drivers of curriculum development, programme evaluation, job function delineation and continuous professional development assessments [7-9]. For some of these purposes, competencies are defined as the “effective application of available knowledge, skills, attitudes and values in complex situations” [7].

Over the past decade, public health competencies have received considerable attention, and have been developed and refined in a range of countries: in the United States of America (USA) they were formulated by the Council on Linkages Between Academia and Public Health Practice in 2001 and revisions adopted in 2010 [10]; the Public Health Agency of Canada published a list in 2007 [11], while in Europe, the Association of Schools of Public Health in the European Region (ASPHER) drafted a list in 2008, which were redefined in 2011 [12]. In the same year (2008), the United Kingdom (UK) Public Health Skills and Career Framework was endorsed [13], while in Australia, the Foundation Competencies for Master of Public Health alumni [14] were published in 2009. These public health competencies were, in many instances, developed through group discussions and a modified Delphi method, with varying degrees of input from academia and public health practitioners at different levels [8,12-14].

In LMICs, the Public Health Foundation of India held a multi-country conference in 2008, attended by a wide range of local and international delegates and experts. Some delegates were commissioned to develop reports on the state of public health training in their own countries, with a view to informing the development of the public health curriculum in India [15]. Since then, public health competencies have also been developed in Latin America [16]. However public health competencies have not been accepted nor validated across LMICs.

Furthermore, although restructuring curricula in terms of competencies constitutes a statement of intent on behalf of the provider, it does not demonstrate whether these competencies have been acquired, nor whether the selected competencies had impact in the workplace or in society. A review by Zwanikken et al. [17] revealed that very few Masters programmes in health and health care have defined their intended impact on the workplace and in society in general, by specifying outcome or impact indicators.

When six institutions offering MPH programmes came together in December 2011 to design a comparative impact evaluation across programmes, each brought to the discussion the set of key competencies which have guided their programmes over the past decade. These were to serve as the basis for formulating the competencies and impact variables against which to evaluate the impact of the MPH programmes across all six institutions involved in this study. As part of the design, these competencies and impact variables were to be validated using a Delphi process. All the institutions were engaged in training health and allied health professionals working in LMICs and included: School of Public Health, University of the Western Cape, South Africa; Hanoi School of Public Health, Vietnam; School of Public Health Fudan, China; National Institute of Public Health, Mexico; University of Medical Science and Technology (UMST), Sudan (through the Ministry of Health), and the Royal Tropical Institute, the Netherlands.

## Methods

The team used a multistep process, starting with the December 2011 meeting of MPH programme convenors from six countries, to reach consensus on a set of public health competencies, develop a list of draft impact variables and design the validation process. The process of developing the competencies and variables aimed to represent the diversity amongst institutional competencies and learning objectives as well to harmonize and streamline the competency statements sufficiently to establish a shared basis for the evaluation. Specific competencies articulated by particular schools were discussed until consensus was reached on whether and how to include them. The resulting set of competencies includes the common competencies of all schools and seeks to articulate key areas of public health performance. The process of modification was discussion, careful deliberation and consensus building during the face to face meeting, email communications and two Skype meetings.

After the competencies were defined and agreed upon, impact variables were developed. The impact variables were divided into impact on the workplace, such as developing improved working procedures within a work unit, and impact on the sector or society, such as improved quality of care for patients. The team formulated the impact variables through inductive logic while taking into consideration the public health competencies that had already been defined. Although these two levels of intended impact are linked, they were not constructed to be directly equivalent.

Prior to embarking on the study, the ethics committees of the six participating institutions, ie the University of Western Cape Senate Research and Ethics Committee, Hanoi School of Public Health Ethic Committee, Fudan University School of Public Health Institutional Review Board, Sudan Medical and Scientific Research Institute (SUMASRI) Ethical Clearance Committee, National Institute of Public Health Ethic Committee, Royal Tropical Institute Research Ethics Committee, granted ethical approval for the study.

The initial meeting was followed by a period of refinement and discussion of the validation design within the team, by email and Skype conferencing. Validation was undertaken using a modified Delphi process. A number of researchers have used the Delphi method to generate consensus on the public health competencies of different health professionals [18-21]. Though the original conceptualisation of the Delphi method includes at least two and sometimes three rounds of feedback by the same experts [21], a modified Delphi process was chosen: this involved consulting a group of experienced public health experts (1st round) invited by each convenor on the basis of maximum diversity, and if need be, a second round by the experts, followed by consultation of a similarly selected group of programme alumni (2nd or 3rd round), and possibly another round with the alumni. The rationale for including alumni was that their experience would enable them to critique the competencies and impact variables from the perspective of what they regard as relevant in their field of work. Maximum professional, gender and cohort diversity criteria were used in their selection.

The five public health experts from each country (N = 31) were asked to review and validate the public health competencies and impact variables using a Likert scale graded from 1 (signifying that the competence was of ‘poor’ relevance) to 5 (indicating ‘excellent’ relevance to the field of public health practice). The intention was that the decision to undertake further rounds of consultation with the same groups should be based on the degree of consensus found. Qualitative comments and suggestions were also invited.

Responses were entered and stored in Microsoft Office Excel® 2003 (Microsoft Corporation, USA), and calculations were made using Excel. Since the results from the first round showed considerable consensus, a further round was not deemed to be required. The experts’ feedback, however, guided further refinement of the competencies and impact variables, which was then circulated to alumni (N = 30) across the six MPH programmes for further validation. Once again, based on the level of consensus in the results of the graduate round, a further round with them was not deemed necessary.

No agreement exists in the literature on how to measure consensus; measures of central tendency and measures of dispersion are often used [22]. According to Argyrous [23], the median can be used with ranked data (ordinal and interval/ratio), but this is not considered useful for scales with few values; in addition the mean can be used for data that are not skewed. Initially we determined the cut-off points as a mean < =3.9 or a variation coefficient > =0.26. However, the data appeared to be skewed, so the median was chosen: a median of 4 or 5 was considered a good degree of consensus.

### Developing the public health competencies and impact variables

The competencies and impact variables which were validated in the Delphi process had been developed through the deliberations of the six LMIC schools of public health. At an early stage, the competencies were compared with those of the Council on Linkages Between Academia and Public Health Practice [10], as these competencies have been widely used elsewhere as a basis for curriculum design [24-26]. Having considered this framework, the team decided to structure the list of competencies into seven competency clusters. Taking into account the argument against ‘atomization’ or fragmentation into component parts of competencies/outcomes, and the recognition that the overarching competence is often considered the best expression thereof [27,28], the team used the clusters to condense and clarify the competencies. In the course of this clustering process, we decided to group ‘analytical/assessment skills’ with ‘public health sciences skills’ , as we consider ‘analytical/assessment’ skills to be embedded in, and the active component of ‘public health skills’.

**Figure 1:**
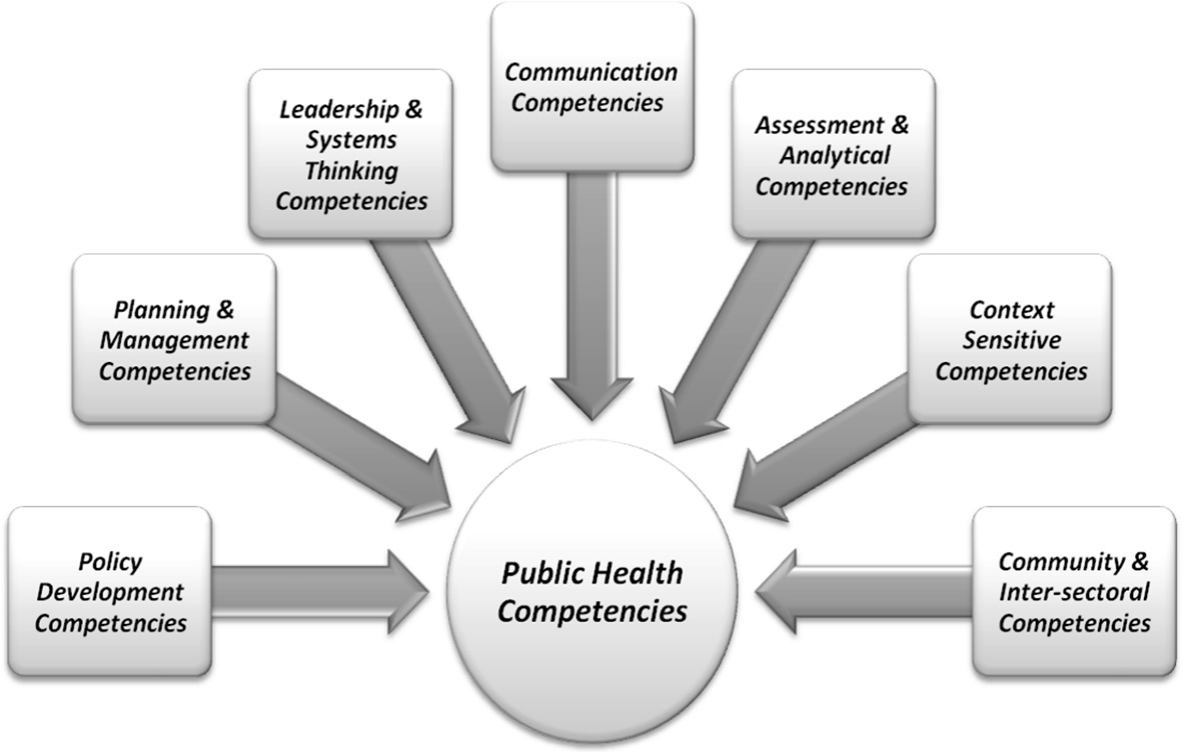
Public health competencies.

In making this comparison, additions to our competencies are notable because we view them as important in the LMICs context. Gender issues were absent from the Council on Linkages framework. The ‘pro-poor and equity-based approach’ was specifically added in response to population needs of LMICs. Furthermore, the team added competencies related to the social determinants of health, as they are considered to be an important foundation for public health practice, as acknowledged in the work of the WHO Commission on the Social Determinants of Health, and others [29]. The conceptualization of cultural competencies was also expanded to encompass ‘context-sensitive competencies’ , in recognition that health status is determined by far more than cultural or background factors, including social, economic, political and gender factors. ‘Policy advocacy’ was added to the domain of policy, as well as the need for ‘context sensitivity’ of policies. ‘Intersectoral competencies’ were added to community competencies, because intersectoral engagement is regarded as a critical principle in furthering the impact of public health. A further contrast with the Council on Linkages framework is that where they assign an important role to financial planning and management, this group’s competencies emphasize planning and management as a whole, including finances (Figure 1).

## Results

As described in the Methodology, the competencies and impact variables were validated using two Delphi rounds, yielding quantitative and qualitative results.

### Validation by experts

To ensure a maximum variation sample of experts in the public health field, the following criteria were used: reviewers should have a broad view on public health, at least 10 years’ work experience in public health, and work at different workplace types. The expert group was required to include both men and women, at least one person from a university (e.g. professor/programme trainer/policymaker of an educational department), one from health system management (i.e. at national/provincial level), one from a service delivery institution (e.g. Centers for Disease Control and Prevention or other public health institutions) and one from a non-governmental organization (NGO). Respondents were recruited by the MPH convener of the respective schools by email or telephone.

Respondents were provided the competencies and impact variables and asked to rate the relevance of each competency and impact variable using a Likert scale from 1 (‘poor’ relevance) to 5 (‘excellent’ relevance’). The key to the competencies and impact variables noted: ‘*Relevance* in this study means that this particular competency is expected of a Public Health Masters graduate working in the field of Public Health’. As regards impact variables: ‘excellent relevance’ suggested that this effect on the graduate’s workplace or sector of society was very important, e.g. they were asked to rate the relevance of ‘Contributed to equity/pro-poor orientation towards health access at all levels’. Comments and additional suggestions were also received from the expert group.

Responses were received over two months from 31 experts (21 men and 10 women). Eighteen of the experts were from universities, seven from health system management, three from service delivery institutions and three from NGOs, including international agencies such as the United Nations Family Planning Association. All public health experts had more than 10 years of experience in public health. In the analysis of data, medians were calculated for all scores as well as for each country to identify cross-country variability (see Additional file 1).

Quantitative analysis across all six countries revealed that 11 of the competencies had a median of 4, while 12 competencies had a median of 5, which shows a high degree of consensus. Qualitative comments focused mainly on the formulation of selected competencies, either pointing out vagueness of expression, a dual focus question or adding to and improving formulations.

Results for the workplace impact variables also showed a high level of consensus: 19 of them had a median of 4, two had a median of 4.5, and three had a median of 5 (Table 1). Two of the lowest variables (with a median of 3) were: the graduate had ‘published book chapters’ , which was considered too high an expectation for an MPH graduate; and ‘Projects [were] rewarded and by what amount’; this was also considered too ambitious and concern was expressed that raising funds could often not be attributed to one person alone. Some experts commented that several workplace variables required a scale, that some were difficult to measure, some too broad and some too ambitious, given certain contexts.

**Table 1:** Results of two Delphi validation rounds

**Round\median**	**Median 3**	**Median 4**	**Median 5**	**Total**
**Round 1 (experts)**				
Number of competencies		11	12	23
Number of impact variables work	2	19	5	26
Number of impact variables society		9		9
**Round 2 (alumni)**				
Number of competencies		10	13	23
Number of impact variables work		19	7	26
Number of impact variables society		7	3	10

All nine of the impact variables on society had a median of 4 (Table 1) across the six countries. General feedback by some experts was that it was difficult to score these variables, as the scoring would depend on the context in which a graduate works. The importance of each variable would depend on the level and role of the graduate, as well as the specific field in which they work, for example as a policy maker, implementation manager, educator or researcher. One expert commented that it would be difficult to attribute an indicator to one person, as so often public health workers operate in teams. Qualitative comments suggested that some society level impact variables were too broad, and too ambitious given certain contexts; in some cases, clarifications were suggested.

Some cross-country variability was identified, with one school scoring overall lower regarding the competencies and variables. In this school’s survey, four out of 23 competencies had a median of 3, while other schools had only one competency with a median of 3, or none. For the impact variables at work, the same school scored three variables less than 3, while other schools scored one to three variables with a median of 3.

For impact variables on society, there were two schools which scored four variables with a median of 3. The competencies and variables which were scored lower in the one school were also not rated highly in other schools, and were changed.

Based on the quantitative results and the qualitative comments from the experts, 14 of the 23 competencies were reworded to improve clarity (Tables 1 and 2). Two of the workplace impact variables were changed because the median of 3 was low: ‘Achievements which can be attributed to the leadership of the graduate, e.g. individual or organisational awards’ was deleted as it was seen as overlapping with another variable, and the following variable was added: ‘Participated in building a successful partnership’ , based on comments from experts. Eleven of the 26 impact variables in the workplace were reworded, based on qualitative feedback (Tables 1 and 3). Four of the nine impact variables on society were reworded based on the qualitative feedback, and one variable was added, based on comments from experts: ‘Influenced better understanding of public health measures amongst the general population’ (Tables 1 and 4).

**Table 2:** Results of validation of competencies

**Competencies\validation**		**Experts**	**Alumni**
**Cluster of competencies**	**Detailed competencies (as sent to alumni)**	**Median**	**Median**
Public Health science skills including analytical assessment competencies	1. Applies the basic Public Health sciences (including but not limited to biostatistics, epidemiology, environmental health services, health services administration and social and behavioral health sciences) to Public Health policies and programs	5	5
	**2. Appraises scope, function and role of Public Health in relation to local context, health system and other social sectors**	4	4
	**3. Assesses population health status and identifies population health problems, risk factors, related Social Determinants, and determines needs**	5	5
	**4. Commissions and critically interprets research findings and/or develops protocol and collects, analyses and synthesizes reliable and valid data using qualitative and quantitative methods**	5	4,75
Policy development competencies	**5. Analyzes and evaluates policy options and determines feasibility for Public Health policies/programs in diverse community contexts, using appraisal of evidence**	4	5
	6. Participates in developing context sensitive policies and strategic plans and translates them into action	4	4
	**7. Understands and contributes to developing and using mechanisms to monitor and evaluate Public Health policies and regulations**	4	4
	**8. Contributes to advocacy of new and existing health policies to the public health and other sectors**	4	4
Communication competencies	**9. Communicates concisely in writing and orally, in person and through electronic means with linguistic and cultural proficiency and appropriateness**	5	5
	**10. Facilitates and integrates input to Public Health policy and programs from a wide range of individual and organizational stakeholders**	4	4
	**11. Uses a variety of culturally appropriate approaches to disseminate Public Health information with consideration to ethical and confidential issues**	5	5
Context sensitive competencies	12. Analyzes the role of gender, cultural, social, economic, political and behavioral factors in the accessibility, availability, acceptability and delivery of Public Health services and programs	4	5
	**13. Incorporates a Social Determinants of Health approach to Public Health needs**	4	5
Community and inter-sectoral competencies	14. Assesses and engages community actors and communities and their linkages and relationships that affect health in diverse social and cultural situations	4	4
	15. Collaborates in community-based participatory efforts	5	4
	16. Develops and maintains partnerships with key stakeholders, including from different sectors	4	4
Planning and management competencies	17. Uses evidence and good practice to address Public Health policy, planning and management issues	5	5
	18. Plans, implements, monitors and evaluates Public Health interventions, programs, resources, services including input, process, outcome and impact	5	5
	**19. Prepares and contributes to manage and evaluate Public Health information systems, human, financial and logistic resources**	4	4
Leadership and systems thinking competencies	**20. Demonstrates leadership as a manager and in team efforts, and is able to lead in Public Health emergencies**	5	5
	**21. Demonstrates professional judgment and ethical standards in data handling and addressing Public Health issues and diverse opinions**	5	5
	22. Leads with applying the understanding of the interconnectedness and dynamic interactions of the Public Health system	5	4,5
	**23. Continues life-long learning and professional development, and stimulates team to do so**	5	4

*Bold type indicates that the competency was changed after feedback from experts and discussion by the research group.

**Table 3:** Results of validation of impact variables in workplace

	**Experts**	**Alumni**
**Impact variables at workplace (as sent to alumni)**	**Median**	**Median**
1. Created evidence (primary or secondary) for decision-making	5	5
**2. Developed a study or a research proposal**	4,5	5
**3. Reported and made recommendations on population health status or needs**	5	5
**4. Contributed to change in policy at workplace where needed**	4	4
5. Contributed to change in policy at one level higher than work institution	4	4
**6. Participated and influenced working committees for program design or policy formulation at provincial, national or international level**	4	4
7. Published or posted in popular (including electronic) media	4	4
8. Made presentations at conferences	4	4,5
9. Published in peer reviewed publications	4	4
**10. Contributed to writing a published chapter of a book**	3	4
**11. Tutored or taught Public Health professionals, trainees or students in the community**	4	4
**12. Developed, reviewed or commissioned educational or Health Promotion media and materials**	4	4
**13. Planned or implemented community health education courses and workshops**	4	4,5
14. Intervened or worked with a Social Determinants of Health Framework in a way that promotes equity and/or is pro-poor	4	4
15. Collaborated/networked/developed partnerships successfully with other departments than health	4	4
**16. Initiated, sustained and evaluated projects with community participation**	4	4
17. Planned and implemented Public Health interventions, programs or policies based on consultation with stakeholders and using evidence and best practice	4,5	4
18. Implemented performance improvement strategies in response to monitoring and evaluation findings	5	5
**19. Contributed to improvements in human resource management**	4	4
**20. Contributed to improving regular working procedures**	4	4
21. Instrumental in initiating a change within the workplace, or at some level beyond	4	4
22. Contributed to addressing the determinants of health e.g. through planning processes, resource allocation or research	4	5
**23. Raised a project grant**	3	4
24. Contributed to reputation-building of workplace	4	4
**25. Participated in national and international collaboration**	4	4
**26. Participated in building a successful partnership (added)**	0	4
**Achievements which can be attributed to the leadership of the graduate, e.g. individual or organisational awards (deleted)**	4	0

*Bold type indicates that the variable was changed after feedback from experts and discussion by the research group.

**Table 4:** Results of validation of impact variables on society

	**Experts**	**Alumni**
**Impact variables on society (as sent to alumni)**	**Median**	**Median**
1. Contributed to changes in policy or strategy in general	4	5
**2. Contributed to changed guidelines, regulations, ordinances beyond the workplace**	4	4
**3. Contributed to influencing communities, organisations, health sector and other sectors than health**	4	4
**4. Contributed to equity/pro-poor orientation towards health access at all levels**	4	4
5. Contributed to changes in resource allocation for interventions, and research, orientated towards equity and addressing the determinants of health	4	4
**6. Contributed to equitable access to quality services**	4	4,5
7. Contributed to improved Public Health in specific areas related to work context, e.g. improved utilization of services	4	4
8. Contributed to increased resource mobilization for Public Health	4	4
9. Contributed to increased resource mobilization for disadvantaged groups	4	4
**10. Influenced better understanding of Public Health measures amongst general population (added)**	0	5

*Bold type indicates that the variable was changed after feedback from experts and discussion by the research group.

### Validation by alumni

After the researchers reached agreement on the revision, each school sent the competencies and impact variables out to five alumni for a second round of validation. The selection of alumni was based on maximum variation per school using criteria of gender, geographical location, year of graduation (between 2004 and 2010) and workplace type. Respondents were again asked to rate the relevance of each competency on a five-point Likert scale – i.e. whether each competency is expected of an MPH graduate working in public health; the key for scoring was revised for greater clarity, and ranged from 1 (‘Not a key competency’) to 5 (‘Highly relevant’) on the advice of one of the experts. Respondents were also asked to rate the drafted impact variables from 1 (‘Not a key variable’) to 5 (‘Highly relevant’). They were also asked for comments and additional suggestions. It took a further two months to gather feedback from these 14 men and 16 women. One of the alumni graduated in 2004; two in 2005; five in 2006; four in 2007; six in 2008; six in 2009; five in 2010; and one in 2011. Of them, ten alumni worked for higher education institutions, 12 in health system management, seven for service delivery institutions and one for an NGO.

Medians for each country were computed to identify cross country variability. Quantitative results revealed that ten competencies had a median of 4, and 13 competencies a median of 5 (Tables 1 and 2), showing a high degree of consensus (see Additional file 2).

In relation to the impact variables in the workplace, 19 of the 26 variables had a median of 4, and seven had a median of 5 (Tables 1 and 3). Seven of the impact variables in society had a median of 4, and three had a median of 5 (Tables 1 and 4).

As regards to cross-country variability for the competencies, alumni from one school rated two competencies at 3, alumni from two different schools scored five out of 23 work impact variables at 3, while alumni from other schools scored a median of 3 for zero, one or three variables. With regard to the impact variables in society, alumni from two different schools scored two and three different impact variables at 3.

General qualitative feedback suggested that the impact variables were dependent on the actual job or workplace of an MPH graduate, as well as the expectation of a student at the start of a program, which concurred with the feedback from the experts. Qualitative feedback suggested that wording could be more specific in 12 of the 23 competencies, 11 of the 26 impact variables on work and five of the 10 impact variables on society. For example, of the fourth competency, ‘Commissions research’ , two alumni (A15 male, A20 male) commented: ‘Consider adding application of ethical principles’. For the second impact variable on society: ‘Contributed to changed guidelines, regulations, ordinances beyond the workplace’, alumni commented: ‘It’s hard because of the old *thought* about what public health is, but we’re pushing for the change’ (A23, Female) and [it is] ‘not easy to demonstrate’ (A27, Female). (See Additional file 3).

## Discussion

A set of competencies and impact variables of MPH programmes were formulated and validated with public health experts and alumni of the programmes. This is the first time, to our knowledge, that public health competencies have been validated for MPH programmes located in or intended for LMICs, across continents. It was also the first time that impact variables of MPH programmes have been formulated and validated. Although there were some variations across countries, the results show an overall consensus of the 23 public health competencies, 26 impact variables on the workplace and 10 impact variables on society.

The process of competency development has differed across the globe: in the USA as well as in the UK, a large number of experts were involved [8,13], but in both cases, the process was criticized for being too strongly directed by the higher ranks [12]. Another approach was taken by ASPHER in the European region, which involved local employer and workforce representatives [12]. Other recent studies reviewing public health competency formulation surveyed only specific stakeholders such as employers [29], experts [20], academic practitioners and employers, but not alumni [18,19]. Higher numbers of people than were used in this study were sometimes included in the panel [18,30], however, the response rate and the level of consensus was lower. None of the initiatives reviewed in the literature developed impact variables.

Public health is in different stages of development in the different countries. However, in spite of this and other contextual diversity factors, the validation process yielded high consensus. It is possible, however, that if experts and alumni had been asked to prioritize or weight the competencies and impact variables, differences would have become more pronounced [30].

The nature of the Delphi method is qualitative in design and does not seek statistical representativeness in the number of experts invited, but rather attempts to achieve maximum variation of the characteristics of the experts, based on purposeful selection [21]. Future work could engage a larger number of experts or ensure a wider variety of experts.

In this validation process, the ‘public health science skills’ as well as the ‘context sensitive competencies’ received the highest ratings from both experts and alumni: clearly the addition of the ‘context sensitive competencies’ was deemed important in the context of LMICs. Further high scoring competencies were ‘planning and management’ , ‘communication’ as well as ‘leadership’ and ‘systems thinking’ competencies. Slightly lower ratings were assigned to ‘policy development’ and ‘community and intersectoral competencies’. Though still highly rated, the ‘policy development competencies’ and ‘community competencies’ might be less valued because of assumptions regarding the working level and roles played by MPH alumni.

The highest scoring impact variables on the workplace amongst experts and alumni were: ‘Created evidence for decision making’ , ‘Developed a study or research proposal’ , ‘Reported and made recommendations on populations health status or needs’ , as well as the variable ‘Implemented performance improvement strategies in response to monitoring and evaluation findings’. Interestingly the variable, ‘Contributed to addressing determinants of health’ was scored higher by alumni than experts, possibly reflecting the alumni’ current experience in the field as well as recent public health developments emphasising the determinants. As for impact variables in society, there was not much difference between the rating of experts and alumni, except for the first indicator: ‘Contributes to changes in policy or strategy in general’: this was rated higher by the alumni. The highest rated competencies and variables were clearly strongly endorsed and indicated a high degree of consensus.

### Limitations

Although the experts came from four different continents and six countries, there was a high degree of consensus regarding the rating of competencies, though this applied to a lesser extent to the impact variables. It is suggested that consensus might have been promoted by social desirability: although the experts were anonymous to one another, they were selected by MPH programme convenors who had developed the competencies and variables. Intra-rater variability, however, showed scores from 1–5. In addition, no prioritization of competencies was requested which might have elicited even greater social desirability bias. By using selection criteria for experts to ensure maximum variation, this bias was reduced.

The validation by alumni yielded similar results, with increased congruency, i.e. no impact variable with a median of 3, and more with a median of 5. For the graduate respondents, it is possible that social desirability may have influenced the results, as they were informed that the competencies and variables had already been reviewed by experts. However given the fact that there was less consensus regarding the impact variables and intra-rater variability was mostly between 2–5, this was probably not the case. In retrospect, it might have been better to engage the alumni in the first rather than the second round, to avoid this possible risk of bias. The question is, however, whether that would have yielded different results, given the already high consensus in the first round. The fact that the alumni were only invited to participate in the second round created the opportunity to improve the competencies and impact variables before they received them. The selection of alumni may also have been biased, as this was undertaken by MPH programme convenors; this bias was minimized through the use of explicit selection criteria.

The validated impact variables yielded relatively high consensus although less than the competencies. Both the experts and alumni commented that contextual factors, such as the position of the graduate or the level at which the graduate was working, influenced whether these variables could be measured; this was raised as a greater concern for the impact variables on society. Some cross-country variability was identified however, given the small number of experts and alumni per country and the fact that no specific school could be identified as differing markedly from others, and given the otherwise high consensus, these findings were thought not to be material to the study.

## Conclusion

This study contributes to the debates and deliberations on appropriate selection of public health competencies in LMICs. The validation of the competencies and impact variables suggests that public health competencies in LMICs should differ from those in high income countries by placing emphasis on factors which impact on the health of their populations, such as examining the social determinants of health, focusing on context specificity and intersectoral competencies, with less emphasis on financial planning in management.

Inasmuch as this formulation and validation process of public health competencies is understood to be a first initiative and that impact variables for MPH alumni working in LMICs have not previously been developed or at least publicised, the study can be said to have provided a foundation for further refinement, and suggested surprising consensus across countries. Although the social, cultural and political situation and the state of public health development differs considerably between the countries where the six MPH programmes are situated, clear consensus emerged as to what the public health competencies and impact variables should entail.

These public health competencies and impact variables can, therefore, be used to design or evaluate MPH programmes and to assess the competencies of individuals engaged in formal programmes and continuous professional education.

## Abbreviations

A: Alumnus; ASPHER: Association of schools of public health in the European region; MPH: Master of Public Health; LMIC: Low and middle income countries; NGO: Non-governmental organisation; UK: United Kingdom; USA: Unites States of America.

## Competing interests

The authors report no competing interests.

## Author’s contributions

PZ conceived the article. PZ, LA, NTH, QX, LMV, XHY, MCGR, MAW and SN were all involved in the data collection. All authors contributed to data analysis, writing and review. All authors read and approved the final manuscript.

## Pre-publication history

The pre-publication history for this paper can be accessed here:

http://www.biomedcentral.com/1471-2458/14/55/prepub

## Supplementary Material

Additional file 1 Responses experts quantitative. Click here for file

Additional file 2 Responses alumni quantitative. Click here for file

Additional file 3 Responses alumni qualitative and summarized quantitative. Click here for file
