# Supplementary material for: Validation of public health competencies and impact variables for low- and middle-income countries
Source: BMC Public Health. 2014 Jan 20;14:55. doi: 10.1186/1471-2458-14-55 (PMC3899921; doi:10.1186/1471-2458-14-55)
Supplement: Additional file 3 — Responses alumni qualitative and summarized quantitative. [file 1471-2458-14-55-S3.docx]

### IMPACT EVALUATION DELPHI REVIEW: EXPERT REVIEW 2: 5 GRADUATES PER SCHOOL

**TABLE A: KEY COMPETENCIES EXPECTED IN A MPH GRADUATE**

Please indicate your score by typing it in the column indicated.

*Key: “Relevance” is taken to mean: this competency is expected of a Public Health Masters graduate working in the field of Public Health; where 5 signifies “a highly relevant competency for MPH graduates”; and 1 signifies “not a key competency expected of MPH graduates”.

| **CATEGORY OF COMPETENCY** | DETAILED COMPETENCIES | **mean** | **Stand**  **ard dev** | **Var**  **coeff** | **Med**  **ian** | **COMMENTS AND ADDITIONAL SUGGESTIONS**  Please make comments and suggest any missing competencies or variables. | **Fudan comments**  **General:**  NO2:Gategory 2 Policy process should change into policy formulation and implementation ;Gategory 4 Context sensitive competencies are difficult to understand | **Mexico comments** | **South Africa**  **comments** | **Sudan Comments** |
| --- | --- | --- | --- | --- | --- | --- | --- | --- | --- | --- |
| 1. Public Health science skills including analytical assessment competencies | 1. Applies the basic Public Health sciences (including but not limited to biostatistics, epidemiology, environmental health services, health services administration and social and behavioral health sciences) to Public Health policies and programs. | 4,7 | 1 | 0,13 | 5 | (1KIT)This is relevant for MPH all concentrations.  (6KIT) Also communication skills - able to communicate fluently both in written, oral and other ways as necessary.  (2HSPH) MPH students should be equipped with hospital management sciences  (2HSPH) MPH students should be equipped with more knowledge in health human resource management, medicai equipment and facilities management, and health resources management | **NO1：**add Health Economics; **NO2:** Is the Biostatistics or the Health Statistics? there are differences between Biostatistics and Health Statistics. Add the ability of how to finding issues and how to solve them in routine working; **NO3:**add Health Ecnomics. | Very aplicative |  | **S1:** all competences needed are covered except for that skills required for an academic job of public health graduate.  **S2:** Also including occupational health, research methodology and health economics.  **S3:** I think it would be better if those skills are not grouped as they very much differ |
|  | 2. Appraises scope, function and role of Public Health in relation to local context, health system and other social sectors. | 4,3 | 1 | 0,16 | 4 | (1KIT)This is especially relevant for MPH with health policy/health development concentration e.g. ICHD at KIT Amsterdam. | **NO2: It's difficult to** define the contents and scope of Public Health. There are different definations of public health. It not only cover public health services, but also related to medical health delivery system. All the government, community, society, and citizen are involved in public health;  **NO5:**it is too difficult for MPH students in terms of the current training program. | during all the MPH -Health Administration |  | **S3:**What about global context?? |
|  | 3. Assesses population health status and identifies population health problems, risk factors, related Social Determinants, and determines needs. | 4,7 | 1 | 0,13 | 5 | (1K)However, this competency should have been included in point 1.1 above. | NONE | very useful |  | **S3:**This could be linked to epidemiology ??!! |
|  | 4. Commissions and critically interprets research findings and/or develops protocol and collects, analyses and synthesizes reliable and valid data using qualitative and quantitative methods. | 4,5 | 1 | 0,13 | 5 | (1K)But we realize that one-year MPH study only has so much time. Most likely students only have exposure to intro biostatistics and epidemiology methods. However, MPH with biostatistics (or epi) concentration should be able to have this.  (2K): Consider adding application of ethical principles to the collection, maintenance, use and dissemination of data & info  (6K): Also able to apply ethical principles in the collection, maintenance, use and dissemination of data and information. Also applies IT and computer systems in data storage and retrieval strategies | NONE | very useful |  | **S3:** Research is competency that is needed by all health practitioners and not only Public health ….maybe a focus on social or health system research ??!!!  **S4** I think this question is loaded with competences and while all are relevant some carry more wight than others for instance commisioning of research might weigh less than critically interpreting or developing protocols. Too many concepts here, |
| 2. Policy process competencies | 5. Analyzes and evaluates policy options and determines feasibility for Public Health policies/ programs in diverse community contexts, using appraisal of evidence. | 4,3 | 1 | 0,24 | 5 | (1K)These competencies from 2.5 to 2.8 are especially relevant for MPH with health policy/planning concentration.  (6K) Also able to state policy options and write clear and concise policy statements. | **NO5:**These compentences should be strengthened in MPH training in future. | in the whole MPH | #2 Policy Analysis is a crucial skill for a MPH graduate and should be one of the core competencies that an MPH graduate should possess. This will then include the ability to critique policy using relevant data | **S2:**Using appraisal of evidence and community opinion. |
|  | 6. Participates in developing context sensitive policies and strategic plans and translates them into action. | 3,9 | 1 | 0,26 | 4 | (5K) This is more relevant to policy makers at high level of Governments who can be guided by advice from professionals (MPH, PhD e.t.c)  (6K) Also able to translate policy into organizational plans, structures and programs | **NO5:**This is multi-disciplines competence. It seems too difficult for MPH. | in the whole MPH |  |  |
|  | 7. Understands and contributes to developing and using mechanisms to monitor and evaluate Public Health policies and regulations. | 4,2 | 1 | 0,25 | 4 | (1K)The field of health economics/ econometrics has been going towards quantitative impact evaluation (IE). MPH with health policy should have at least intro exposure on IE since this is extensively used by national/international agencies e.g. World Bank, etc. | **NO2:**hard to understand | in the whole MPH |  |  |
|  | 8. Contributes to advocacy of new and existing health policies to the public health and other sectors. | 4,2 | 1 | 0,21 | 4 | (6K) Also able to identify interpret and implement public health laws, regulations and policies related to specific programs | **NO2:**What's the reason of advertising new and existing policies simultaneously. What's the aim of it? | in the whole MPH |  |  |
| 3. Communication competencies | 9. Communicates concisely in writing and orally, in person and through electronic means with linguistic and cultural proficiency and appropriateness. | 4,6 | 1 | 0,15 | 5 | (1K) The competencies in this section 3 are very useful for MPH students in all concentrations (bio, epi, policy, management, etc). However, current public health has been adopting the field of political economy which has its own field/expertise in public health (may be included in MPH in health policy concentration).  (6K) Also exhibit the right attitude such as listening to others in an unbiased manner, respecting the points of view of others and promoting the expression of diverse opinions and perspectives. | **NO5:**It is the needed ability in each field, should haven't been included in MPH training. | in the whole MPH |  | **S2:** Dealing with people from different cultural backgrounds |
|  | 10. Facilitates and integrates input to Public Health policy and programs from a wide range of individual and organizational stakeholders. | 4,1 | 1 | 0,21 | 4 | (6K) Also leads and participates effectively in groups to address specific issues | **NO2:It may be** better if it transfer to the table 2 | In the whole MPH |  |  |
|  | 11. Uses a variety of culturally appropriate approaches to disseminate Public Health information with consideration to ethical and confidential issues. | 4,3 | 1 | 0,21 | 5 |  | **NO5:**Hope more such training in MPH education in future. | In the whole MPH |  | **S3:** How about designing and delivering instead of using ?? |
| 4. Context sensitive  competencies | 12. Analyzes the role of gender, cultural, social, economic, political and behavioral factors in the accessibility, availability, acceptability and delivery of Public Health services and programs. | 4,3 | 1 | 0,2 | 5 | (1K) This section is relevant for MPH graduates from all concentration. Every MPH graduates should have exposure for these two points of 12 and 13. Given the importance of #13 for instance, there could be MPH with SDH concentration.  (5K) 11 seems to be broken down further in 12; could the 2 (11 & 12) be merged?? | **NO2:**need to delete gender, no difference between accessibility and availability; **NO3:**add feasibility | In the whole MPH |  |  |
|  | 13. Incorporates a Social Determinants of Health approach to Public Health needs. | 4,5 | 1 | 0,18 | 5 |  | None | Specifically in the social sciences subjects |  |  |
| 5. Community and inter-sectoral competencies | 14. Assesses and engages community actors and communities and their linkages and relationships that affect health in diverse social and cultural situations. | 4,2 | 1 | 0,24 | 4 | (1K)I still consider this relevant for MPH graduates. Assessing the community actors and identifying partnerships could be taught through stakeholder analysis. But, teaching the competency to engage with the community or maintain partnerships can be a real challenge. If time for educating MPH is limited, there is option to teach them how to collaborate with “social workers” on the field with engagement skills.  (5K) 14 seems to be mirrored in 15. | None | During the communitary pratice |  |  |
|  | 15. Collaborates in community-based participatory efforts. | 4,2 | 1 | 0,19 | 4 |  | None | During the communitary pratice |  |  |
|  | 16. Develops and maintains partnerships with key stakeholders, including from different sectors. | 4,3 | 1 | 0,17 | 4 | (5K) 10 above is a preliminary step in achieving this and both are highly relevant  (6K) Also understands and promotes workable public-private partnerships for effective community interventions | **NO2:**partnerships or collaotation? **NO5:**It's the duty of government rather than public health sector. | During the communitary pratice and thesis development |  | **S2:** Realize the difference between role of governmental and non-governmental organizations. |
| 6. Planning and management competencies | 17. Uses evidence and good practice to address Public Health policy, planning and management issues. | 4,6 | 1 | 0,15 | 5 | (1K)For MPH in health policy/planning concentration (e.g. ICHD at KIT), these competencies are highly relevant. However, such skills requires some exposures in politics, economics, technical (epi and or project management), and ethics.  (6K) Also utilizes current techniques (including cutting edge solutions) in decision analysis and health planning. | None |  |  | S4: With regard to planning and management competencies (6)it is critical to note that a basic understanding is necessary but expertise across all these might not be necessary eg one who plans and implements might not necessarily have to monitor and evaluate if there are people with that expertise as is sometimes the case. My concern hear is that if this impact evaluation finds that all these competencies are critical will it mean curricula need to be revised so that MPH students are able to understand and do all these? Is that kind of program possible? Or would it be acceptable for them to have a basic understanding of some subjects and expertise in others?. |
|  | 18. Plans, implements, monitors and evaluates Public Health interventions, programs, resources, services including input, process, outcome and impact. | 4,7 | 0 | 0,1 | 5 |  | **NO2:**delete 'interventions','programs' and 'resources'; **NO5:**expecting more such training in MPH education in future. | This is very helpful if you take an addition diploma in Health Evaluation |  | **S2:** And to be able to use the M&E to improve program/project performance. |
|  | 19. Prepares and contributes to manage and evaluate Public Health information systems, human, financial and logistic resources. | 4,3 | 1 | 0,2 | 4 |  | None | Reinforcement during the Management Subject-matter |  | **S2:** It is important to be able to run projects even when there are financial constraints.  Manage to resolve conflicts in workplace |
| 7. Leadership and systems thinking competencies | 20. Demonstrates leadership as a manager and in team efforts, and is able to lead in Public Health emergencies. | 4,3 | 1 | 0,24 | 5 | (1K)While many would agree that MPH graduates from all concentrations should ideally have these competencies, there might not be enough study time to teach all these to MPH students with more technical skills such as MPH in biostatistics or epidemiology concentration. | **NO5: Students may not be the manager or leader after awarding MPH degree. So** leaderships may be selective ability. | Reinforcement during the dissasters topics | #1 suggests we add competencies 24 and 25: Shows an enhanced capacity in respect of sensing the need for and understanding the nature of change (in leadership) and the need to deal with this change as an innovative leader i.e. Addition to include understanding and practice of leadership for learning and innovation; and  #1 Shows an enhanced capacity in respect of an improved understanding of self and the impact of self on innovative leadership and improved entrepreneurial capacity, i.e. Addition to include/enhance understanding of the self, creativity and entrepreneurship. |  |
|  | 21. Demonstrates professional judgment and ethical standards in data handling and addressing Public Health issues and diverse opinions. | 4,4 | 1 | 0,2 | 5 | (1K)So for the competencies in this section, effort to teach these competencies is important especially for MPH in health policy/management concentration. | **NO5:**It's related to professional value, personality, and ethics. However, personality is not directly connected to training. There are many determinants on personality, such as contexts, social value and so on. | Reinforcement during the Management Subject-matter |  |  |
|  | 22. Leads with applying the understanding of the interconnectedness and dynamic interactions of the Public Health system. | 4,3 | 1 | 0,17 | 5 | (6K) Also understands the historical development, structure and interaction of public health and health care systems | **NO3:**delete 'dynamic' | During the MPH | #2 Understanding the complexities of systems, interlinks and interactions is a crucial leadership competency |  |
|  | 23. Continues life-long learning and professional development, and stimulates team to do so. | 4,3 | 1 | 0,17 | 4 |  | **NO3:**change simulate into lead; **NO5:**not necessary | Very useful |  |  |

**S4**:

**A BRIEF INTRODUCTORY GUIDE IS IMPORTANT HERE. SEE PREVIOUS COMMENT ON WHOSE PERSPECTIVE TO RESPOND.**

**I am not sure about this** 1 = Not a key variable

2 = Some relevance

3 = Average relevance

4 = Above average relevance

5 = Highly relevant

why does 1 have to be not a key variable???? It does not seem like a good likert scale because the rest of the variables are looking at relevance while this one is checking whether it’s a key variable or not. I would propose to use something along the lines of irrelevant or not relevant!!!!

| **TABLE B:** **IMPACT VARIABLES AT THE WORKPLACE** | **mean** | **Stan**  **d dev** | **Var**  **Co**  **eff** | **Med**  **ian** | **COMMENTS AND ADDITIONAL SUGGESTIONS**  (3K) Variables or Indicators? And if so, do they need to be more SMART? If they need to be measured/verified more precision may be needed while defining them? | **Fudan comments** | **Mexico comments** | **South Africa comments** | **Sudan Comments** |
| --- | --- | --- | --- | --- | --- | --- | --- | --- | --- |
| 1. Created evidence (primary or secondary) for decision-making. | 4,5 | 0,6 | 0,13 | 5 | (1K) is not clear for me whether these impact variables are those that are (ideally) expected from MPH graduates or those that are (actually) personally experienced as MPH graduate. I filled in with the latter.  (3K) Evidence with PH implications. Good if happens but not all PH professionals will be in position to generate knowledge/evidence  (6K) May also contribute to the creation of evidence as part of a team. | None | You get the skills to look for the evidence |  | **S2:**Contribute to build a public health research data base  **S3:** What is the deference between 1 & 2? (isn’t the purpose of the study is evidence ??) |
| 2. Developed a study or a research proposal. | 4,5 | 0,6 | 0,14 | 5 | (3K)Research or study proposal with PH implications  Good if happens but not all PH professionals will be in position to generate knowledge/evidence | **NO2**:change 'or' into 'and' | During the MPH you accquire the skills |  | **S2:** Critically appraise and identify limitations in research findings |
| 3. Reported and made recommendations on population health status or needs. | 4,8 | 0,6 | 0,12 | 5 | (3K) Recommendation based on appraisal of evidence (existing or new); comparing available options. Essential role of PH professionals. Consider splitting the situation analysis from the recommendation part into 2 different variables? | **NO2**:develope such things | You have good skills to make reocmendations |  |  |
| 4. Contributed to change in policy at workplace where needed. | 4,1 | 0,9 | 0,23 | 4 | (3K) Recommendation based on appraisal of evidence (existing or new); comparing available options. Essential role of PH professionals. Situation analysis missing? | **NO2**:Good policy needn't change; **NO5:**It's a long time to make change | Alumni from INSP are well recognized and take into consideration for new policies by instance at my workplace. |  | **S3:** Sometimes very difficult to demonstrate |
| 5. Contributed to change in policy at one level higher than work institution. | 3,8 | 1,1 | 0,27 | 4 | (3K) Recommendation based on appraisal of evidence (existing or new); comparing available options. Good if happens but not all PH professionals will be in position to do it | **NO2:**not every policy needed to change, Good policy needn't change | We're asked to collaborate with other institutions |  | **S3:** Even more difficult |
| 6. Participated and influenced working committees for program design or policy formulation at provincial, national or international level. | 4,1 | 1,0 | 0,25 | 4 | (3K) Essential role of PH professionals | **NO2: It** should also be in district level; **No5:** We were involve in but cannot make decision. | Now I'm participatind at provincial level. |  |  |
| 7. Published or posted in popular (including electronic) media. | 4 | 0,9 | 0,21 | 4 | (3K) Good if happens | None | That's a goal after finishing the MPH |  | **S3:**Could this be replaced by any sort of published reports ?? |
| 8. Made presentations at conferences. | 4,2 | 1 | 0,24 | 4,5 | (3K) Essential role of PH professionals  (5K) Depends on opportunity; but an MPH graduate must be able to do this | None | I had the opportunity to present in Brazil, Canada and Emory Atlanta. | #2 Presentations at conferences are ideal for those in academia etc however there are other platforms where graduates may present their work such as symposiums or seminars. Therefore this variable should include the other platforms | **S3:**Of original work ?? |
| 9. Published in peer reviewed publications. | 3,8 | 1,2 | 0,31 | 4 | (3K) Good if happens  (5K) Several factors determine publications e.g. publishers, editors, reviewers, perceived relevance, e.t.c | None | My first article was adminted 6 months after I submited to the WHO bulletin |  | **S3:**This looks like no (7) |
| 10. Contributed to writing a published chapter of a book. | 3,6 | 1,2 | 0,33 | 4 | (3K) Good if happens | None | I'm working in a chapter |  |  |
| 11. Tutored or taught Public Health professionals, trainees or students in the community. | 4,1 | 1,0 | 0,22 | 4 | (3K) Essential role of PH professionals | None | I'm teaching medical students in the MPH field as well as quality improvement | #2 Graduates may serve as mentors in their workplaces |  |
| 12. Developed, reviewed or commissioned educational or Health Promotion media and materials. | 3,9 | 0,8 | 0,21 | 4 | (3K) Good if happens  (5K) There are existing specialists in this area who could be MPH holders but with additional training on IEC/ACSM | **NO2:**hard to understand | I've developed educational materials |  |  |
| 13. Planned or implemented community health education courses and workshops. | 4,3 | 0,8 | 0,19 | 4 | (3K) Good if happens | **NO2:**hard to understand | I'm planning to start community approach in the following term with medical students |  |  |
| 14. Intervened or worked with a Social Determinants of Health Framework in a way that promotes equity and/or is pro-poor. | 3,7 | 1,1 | 0,29 | 4 | (3K) Essential role of PH professionals | **NO2:**Does that mean equality of basic public health services? **NO5:**It's necessary but hard to achieve it. | I'm collaborating in a CONACYT equity research project |  |  |
| 15. Collaborated/networked/developed partnerships successfully with other departments than health. | 4,1 | 0,9 | 0,21 | 4 | (3K) Essential role of PH professionals | None | I'm collaborating within a NGO in San Luis Potosi |  |  |
| 16. Initiated, sustained and evaluated projects with community participation. | 3,8 | 1,2 | 0,31 | 4 | (3K) Essential role of PH professionals | **NO2:**Initiated, sustained and evaluated projects should not implemented by one person. **No5:** It's competence in my part-time volunteer service delivery. | Not yet |  | **S2:** And Participatory research  **S3:** Not only with community but all relevant partners and delivered FEEBACK |
| 17. Planned and implemented Public Health interventions, programs or policies based on consultation with stakeholders and using evidence and best practice. | 4,3 | 0,7 | 0,17 | 4 | (3K) Essential role of PH professionals | None | At the General Hospital in San Luis Potosi, I'm working in a rehabilitation project |  |  |
| 18. Implemented performance improvement strategies in response to monitoring and evaluation findings. | 4,5 | 0,6 | 0,13 | 5 | (3K) Essential role of PH professionals | None | We impemented "Shadow studys" to monitoring clean hands |  |  |
| 19. Contributed to improvements in human resource management. | 3,9 | 0,9 | 0,22 | 4 | (3K) Essential role of PH professionals | **NO5:** This contribution are from leader. | At the General Hospital in San Luis Potosi. |  |  |
| 20. Contributed to improving regular working procedures. | 3,9 | 0,9 | 0,23 | 4 | (3K) Good if happens | None | I used the "Fish Bone" diagram to improve process |  | **S3:** SOPs?? |
| 21. Instrumental in initiating a change within the workplace, or at some level beyond. | 4 | 1 | 0,25 | 4 | (3K) Better defined? What kind of change? | **No5:** It's not hard to initiati a change, but it is hardly applied. | We are initating the Quality and Patient Safety Branch at the General Hospital in San Luis Potosi |  | **S3:** How about contributed ? |
| 22. Contributed to addressing the determinants of health e.g. through planning processes, resource allocation or research. | 4,3 | 0,9 | 0,21 | 5 | (3K) Essential role of PH professionals | **NO2:**delete "research" | Not as primary duty right now. |  |  |
| 23. Raised a project grant. | 3,8 | 1,0 | 0,26 | 4 | (3K) Wrote and submit a project grant? This implies situation analysis, appraisal of evidence and comparison of options available taking into consideration the local context + being aware of trends and tendencies in ‘development world’  (5K) Limited successes; but should be able to prepare a sound application for the grant | None | Not yet |  |  |
| 24. Contributed to reputation-building of workplace. | 4 | 0,8 | 0,21 | 4 | (3K) Better defined? | None | We want to certificate the General Hospital |  | **S3:** Subjective !! |
| 25. Participated in national and international collaboration. | 3,7 | 1,1 | 0,3 | 4 | (3K) Good if happens | **NO2:**for public health, it is better to focuse on local level. National and international level seems far away from our working. | Not at this momment |  | **S3:** Maybe to define the participation more |
| 26. Participated in building a successful partnership. | 3,9 | 1,1 | 0,27 | 4 | (3K) Essential role of PH professionals | **NO2:**need to redefine "partnership" from degree and scope aspect. ;**NO3:**Who are partners? | We're collaborating with the School of Medicina and other NGO's. |  | **S2:** Participated and maintained |

| **TABLE C: IMPACT VARIABLES ON SOCIETY** | **mean** | **stdev** | **Var**  **coeff** | **median** | **COMMENTS AND ADDITIONAL SUGGESTIONS** | **Fudan comments** | **Mexico comments** | **Sudan Comments** |
| --- | --- | --- | --- | --- | --- | --- | --- | --- |
| 1. Contributed to changes in policy or strategy   in general. | **4** | 1,1 | 0,26 | 5 | (3K) Better defined? Outside the health sector? | **NO2:**change "changes"into "improvements and perfection" | I'm collaborating to write new Hospital policies | **S3:**Maybe developing or updating ?? |
| 2. Contributed to changed guidelines, regulations, ordinances beyond the workplace. | 3,8 | 1,1 | 0,29 | 4 | (3K) Better defined? Outside the health sector? | **NO2:** changes in regulations only happen though legal procedures; **No5:** It's not evidence-based policy making in current public health. Community actors were not involved in policy formulation, and also were hard to contribute policy change. These issues were related to governance, administration model and democracy improvement. | It's hard because of the old "thought" about what Public Health is, but were pushing for the change. | **S3:**Not easily demonstrated and I think it represents lack of focus |
| 3. Contributed to influencing communities, organisations, health sector and other sectors than health. | 3,9 | 1,1 | 0,27 | 4 | (3K) Better defined? | **NO2:**add NGOs | Not at this point | **S3:**Similar to partnerships ?? |
| 4. Contributed to equity/pro-poor orientation towards health access at all levels. | 3,9 | 1 | 0,25 | 4 | (3K) Better defined? | None | Not at this point | **S3:**Difficult to achieve and assess in LMICs |
| 5. Contributed to changes in resource allocation for interventions, and research, orientated towards equity and addressing the determinants of health. | 4,1 | 1,2 | 0,29 | 4 | (3K) To table B? | **NO5:** Resouce allocation is very complicated. Public health sectors is litter powerful in it. | Not at this point | **S3:**Why only (Equity??) |
| 6. Contributed to equitable access to quality services. | 4,2 | 1 | 0,24 | 5 | (3K) To table B? | **NO2:**equity and quality are always contradictive | I'm working in the Quality and patient Safety Branch | **S3:**How ?? |
| 7. Contributed to improved Public Health in specific areas related to work context, e.g. improved utilization of services. | 4,2 | 1 | 0,24 | 4 | (3K) Essential role of PH professionals | None | Working on improving patient Safety and Helath Quality Services | **S3:**How ?? |
| 8. Contributed to increased resource mobilization for Public Health. | 3,9 | 1,2 | 0,32 | 4 | (3K) Better defined? Outside the health sector? | **NO5:**It's determined by government, not MPH students | Not at this point | **S3:**Advocacy activities in general |
| 9. Contributed to increased resource mobilization for disadvantaged groups. | 3,7 | 1,1 | 0,31 | 4 | (3K) Better defined? Outside the health sector? | None | Not at this point |  |
| 10. Influenced better understanding of Public Health measures amongst general population. | 4,3 | 0,9 | 0,22 | 5 | (3K) Essential role of PH professionals | None | Not at this point |  |
